# Users' Perspectives on the Organization of Rehabilitation Services – A Focus Group Study of User Organization Representatives in Norway

**DOI:** 10.1111/hex.70139

**Published:** 2024-12-25

**Authors:** Helene Lundgaard Søberg, Per Koren Solvang, Nada Andelic, Cecilie Røe, Marit Kirkevold

**Affiliations:** ^1^ Department of Physical Medicine and Rehabilitation Oslo University Hospital Oslo Norway; ^2^ Faculty of Health Sciences Oslo Metropolitan University, OsloMet Oslo Norway; ^3^ Research Centre for Habilitation and Rehabilitation Models and Services (CHARM), Institute of Health and Society, Faculty of Medicine University of Oslo Oslo Norway

**Keywords:** health services, patient advocacy, patient participation, patient representatives, rehabilitation

## Abstract

**Background:**

User organizations for people with disabilities in Norway work for social equality and participation, and quality of health services for people with disabilities, chronic illnesses and reduced functional capacity. Consideration of the experiences from user representatives is necessary when determining the quality and appropriateness of the rehabilitation services. Rehabilitation services constitute the provision and delivery of intangible products to maintain or improve functioning in individual patients or patient groups. Rehabilitation services can be characterized at the policy (macro), organizational (meso) and individual (micro) levels.

**Objectives:**

To explore user representatives' perspectives on rehabilitation service provision and organization and how they experience the influence they exert.

**Methods:**

Focus group interviews with 14 representatives nominated from 11 user organizations in Norway conducted in 2021. Two online focus groups using a semi‐structured interview guide were conducted. Data analysis was performed according to Braun and Clarke's thematic data analysis.

**Results:**

Six core themes were developed when analyzing the participants' experiences and opinions regarding rehabilitation services. The themes were inter‐connected and addressed perspectives on Access to services, Integration of care, Rehabilitation team, Person centeredness, System and governance and Modes of user representation and contribution.

**Conclusion:**

The user representatives revealed tension and complexity influencing the provision and organization of rehabilitation services from individual access to health policy and regulation. Empowering user representatives through training was important to fight tokenism. Filling the role of a user representative at the meso level requires the integration of personal and peer experiences at the micro level, and knowledge of health policy regulations at the macro level.

**Patient or Public Contribution:**

The Norwegian Federation of Organisations of Persons with Disabilities recruited user representatives in this study. The user representatives participated in the assessment and discussion of the results of the study. The results were presented for discussion to the User panel at the Research Centre for Habilitation and Rehabilitation Models & Services (CHARM) at the University of Oslo.

## 1 Background

The World Health Organization (WHO) defines rehabilitation as ‘a set of interventions designed to optimize functioning and reduce disability in individuals with health conditions in interaction with their environment’ [1]. The organization of rehabilitation services comprises the provision and delivery of intangible products to maintain or improve functioning in individual patients or patient groups and should be provided at multiple administrative and organizational levels, including both specialized and municipal services [2]. These services are steered at the policy (macro) level, carried out at the organizational (meso) level and consumed at the individual (micro) level [3]. Strengthening rehabilitation in health systems requires the involvement of numerous stakeholders at all organizational levels, including user representatives [4, 5, 6, 7].

Solvang et al. describe the three interconnected perspectives on rehabilitation in a social context for the users [3, 8]. At the micro level, users make everyday life decisions relevant to their rehabilitation and care provision; at the meso level, users act as representatives in advisory bodies in hospitals and municipalities; and at the macro level, users are identified as key agents, acting as advisory bodies and advocacy groups impacting health care policy and systems.

Related to rehabilitation service delivery, the importance of user participation in healthcare is emphasized [9, 10]. On an individual level, shared decision‐making in goal‐setting processes should be standard procedure, and involvement in the identification of preferred endpoints and outcome measures is a recognized approach to the co‐creation of new knowledge in research. At the policy (macro) and administrative (meso) levels of rehabilitation services, users are entitled by law to a voice. Thus, rehabilitation service users and their organizations should have a role in the organizing of rehabilitation services and rehabilitation research [11, 12]. A way of including user representatives at the meso level is to appoint representatives from disability organizations to hospital boards, municipal advisory bodies and research projects.

User organizations in Norway work for social equality and participation for people with disabilities, chronic illnesses and reduced functional capacity. The Norwegian Federation of Organisations of Persons with Disabilities is an umbrella organization with 88 member organizations [13] that actively promotes enhanced living standards and rights for people with disabilities and their families. Its activities include lobbying governmental bodies, providing welfare law advice, and advocating in areas like health, accessibility education and employment.

In their scoping review Olsson et al. stated that user representatives who participate on the meso‐ and macro levels play an important role in the development of health services [14]. In Norway, the involvement of user representatives is a widespread practice. One effect is that this has enabled disability organizations representing rehabilitation service users to become highly competent in advising stakeholders on the best way to organize rehabilitation services [15, 16]. However, there is a paucity of studies addressing service user representatives at the meso level in rehabilitation research [14]. There is, thus, a need for additional knowledge of how representatives from the service user organizations for people with disabilities experience and contribute to the rehabilitation systems and services. The current study gives voice to the user organization representatives as stakeholders within rehabilitation systems, services and provision in the context of an ongoing international research project on the organization of rehabilitation services, the International Classification of Service Organization – Rehabilitation (ICSO‐R) [17, 18, 19].

The aim of this study was to generate knowledge regarding the perspectives of user organization representatives on rehabilitation service provision and organization. Specifically, we sought to explore the representatives' perspectives on rehabilitation services at the meso level.

## 2 Methods

The study had a qualitative design that employed focus group interviews of representatives nominated from service user organizations in Norway in 2021. Data collection was based on a semi‐structured interview guide (Table 1) focusing on the quality, provision and organization of rehabilitation services. The interviews were conducted online by authors H.L.S. and M.K. The participants were informed about the interviewers' professions as a nurse and a physical therapist, both with extensive experience in rehabilitation research. The interviews were sound recorded, transcribed verbatim and stored on a secure research server at the OUH. The COnsolidated criteria for REporting Qualitative research (COREQ) were applied to ensure the quality of this study [20].

**Table 1:** Semi‐structured interview guide.

What are the most important factors that contribute to good quality in rehabilitation services? What are key factors that supported your rehabilitation, and what are those that were barriers in the services? Are there particular factors/characteristics with regard to the organization of the service that impact the quality and relevance for rehabilitation? How important is it that users/patients are involved in organizing rehabilitation services? Do you have any thoughts about aspects of organizing rehabilitation services where user involvement is especially important? How should user involvement be organized to ensure sufficient impact on the services provided? What are the major issues that need to be addressed by the user organizations to ensure consistently good and relevant services?

### 2.1 Setting, Recruitment and Participants

Rehabilitation is provided on specialized, intermediate and primary health care levels in Norway. The Norwegian system represents the Scandinavian welfare model with public access to state‐funded rehabilitation services independent of work status or insurances [21]. Organizations of people with disabilities are recognized as stakeholders at the meso and macro levels [22, 23, 24].

This study was organized by the Research Centre for Habilitation and Rehabilitation Models & Services (CHARM) at the University of Oslo, a collaborator in the development of the ICSO‐R [17, 25]. An invitation to participate in the focus group study was sent to all 88 member organizations of the Norwegian Federation of Organisations of Persons with Disabilities. Fourteen representatives from 11 main‐ and local organizations or sub‐divisions agreed to participate, see Table 2. Participants received oral and written information regarding the study, and written consent was obtained from all participants. Two focus group interviews were conducted on Zoom with six and eight participants, respectively, with each lasting about 1.5 h. The participants had their cameras turned on during the interviews.

**Table 2:** Information on the 14 focus group participants.

Sex	
Male	6
Female	8
Mean Age (SD) (range) (*n* = 13)	63.5 (9.7) (43–79)
Education, *n* (%) (*n* = 13)	
Highschool	3
University	10
Years experience with rehabilitation Median (IQR) (*n* = 13)	17.5 (10.8–38.3) Range 5–60
Years of experience as a user representative	8.5 (6.3–17.5) Range 0–40
Median (IQR) (*n* = 12)	
Organization:	
Umbrella organization for people with disabilities	2
Associations for people with Musculoskeletal and Rheumatic disorders	5
Organization/Association for people with Neurodegenerative Disorder	2
Association for people with Sensory Disability	1
Associations for people with Cardiovascular and Respiratory disorders	3
Association for the Traumatically Injured	1

Abbreviations: IQR, Interquartile range; SD, standard deviation.

### 2.2 Analysis

Data analysis was guided by Braun and Clarke's approach to reflexive thematic analysis by identifying and interpreting patterns across the transcribed interviews [26, 27]. We followed the six steps outlined by Braun and Clarke which include becoming familiarized with the data, generating initial codes, searching for themes, reviewing themes, defining and naming the themes, reporting the findings [26]. All steps in the analysis were performed by authors H.L.S. and M.K., and author P.K.S. became involved from the step of reviewing of themes. Through reading and rereading the transcribed text, we familiarized ourselves with the data. In each of the transcribed interviews, data segments in the form of text passages that were relevant to the provision and organization of rehabilitation services were marked, and initial codes were generated [17, 18].

The initial codes and their relevant text passages were collated under thematic headings. Initial core themes relating to the research aim and question were generated based on clusters of similar codes describing issues and topics emphasized by the user representatives. The themes were reviewed and further developed with subthemes based on the codes and the data set as a whole. The reflexive thematic approach underlines that themes are created by researchers actively reflecting on and interpreting the data by generating codes and constructing themes [27]. Theme labels were then created. Quotations from the interviews in which the participants were given fictional names were translated from Norwegian to English by author H.L.S.

Based on a previous study in which user representatives incorporated both personal experiences and policy issues into their discussion of rehabilitation topics [28], we applied the micro–meso–macro perspective as a sensitizing strategy in the analysis process to enable a clearer take on how the levels interact.

To strengthen the validity of the results and enhance user participation in the study, we presented the preliminary core themes and subthemes for discussion in an online meeting, which was attended by eight of the user representatives. The representatives recognized the themes and provided additional details to strengthen the findings, though no new themes emerged.

### 2.3 Ethics

The study was approved by the Norwegian Agency for Shared Services in Education and Research approval # 863072 and conducted according to the Declaration of Helsinki. The participants provided their written informed consent. Before the focus group interviews, author MK informed the participants regarding the purpose of the study, the importance of all participants having a voice when discussing the topics in the interview guide, and the confidentiality of the interviews. All participants were given the semi‐structured interview guide in advance.

## 3 Results

We identified six core themes anchored on the organization and provision of rehabilitation services voiced by the service organization representatives as essential for the quality of rehabilitation services. The themes were Access to services; Integration of care; Rehabilitation team; Person centeredness; System and governance; Modes of user representation and contribution. The core themes and their sub‐themes are presented in Table 3.

**Table 3:** The core themes and sub‐themes developed from the analysis of the focus group interviews with user organization representatives.

Core theme	Sub‐themes
Access to Services	Specialist versus generalist services Timeliness Priorities
Integration of Care	Cooperation and competence transfer Information flow Randomness or predictability Handling the system
Rehabilitation Team	Competence in the rehabilitation team − Profession specific competence − Interdisciplinarity competence
Person Centeredness	Individual users' treatment and rehabilitation process Personal effort underscored User organizations' integrated person‐centeredness Patient‐centeredness meeting user representatives at an organizational level
System and Governance	Ensuring a system that benefits the users User participation and collaboration in administrative boards and councils The political turn in the organizations' approach to political and legislative processes
Modes of user representation and contribution	User participation and tokenism Training and the organizations responsibility

### 3.1 Access to Services

Rehabilitation should be equally available to everyone with service needs. The user representatives underscored that both specialized rehabilitation services and municipal‐level services are needed depending on the type, severity and trajectory of the disorder or disability. The representatives highlighted that patients with diagnoses most in need should receive highly specialized rehabilitation, while user groups with less severe conditions could be treated at the municipal level. They recognized that while specialized services are provided by professionals with expertise in specific conditions, municipal outpatient rehabilitation services are largely provided by less specialized professionals with a breadth of competence:

Hannah: I am thinking that you cannot say that rehabilitation should be organized at the same level for all diagnoses. One must differentiate and build highly specialized competencies for disorders that require it, while for others, we need to establish more [services] in their home environment.

Receiving the right services at the right time was important both related to disease fluctuations and urgency regarding rehabilitation needs. This was highlighted for all patient groups. However, timeliness in the provision of specialized rehabilitation for children and their families in a home‐based context was seen as particularly important as delayed rehabilitation can negatively impact school and social activities, and siblings can also be affected and require support.

One user representative questioned the change in health policy that has restricted access to specialized institutional rehabilitation for people with musculoskeletal disorders who are now typically referred to the municipalities that provide generalist instead of specialized services. The participants additionally expressed concerns that access to institutional rehabilitation is often limited unless returning to work is the expressed goal. They pointed out that users could substantially benefit from institutional rehabilitation even if returning to work was not a viable goal.

### 3.2 Integration of Care

Patients' rehabilitation trajectory from a specialized administrative level to the municipal service level requires integrated organizational systems to ensure an effective flow of information between services regarding patients' rehabilitation processes. The need for a seamless coordination and continuity in the rehabilitation process among institutions and organizational levels was emphasized.

Hannah: If you have been there [in a specialized rehabilitation institution], getting follow‐up at your place of residence afterward and continuing the treatment measures there is a challenge today. Competence transfer, critical documentation, what is written and sent out [to the municipal services] varies a lot.

Information appeared to be a keyword for the integration of care. The lack of information transfer that could facilitate access to services and personnel with special competence in primary health care was described as a barrier to the continuity of qualified rehabilitation. Furthermore, navigating the administrative system and functioning as a rehabilitation coordinator on their own behalf when the system fails to accommodate their service needs was perceived as time‐ and energy‐consuming.

### 3.3 Rehabilitation Team

The user representatives emphasized the importance of receiving high‐quality treatment and voiced a threefold understanding of professional competence. First, the professionals should be highly qualified in their profession, as for example as physical therapist. Second, the users emphasized the need for professionals with specialized competence in treatment of the specific diagnosis groups they represented. They had experienced that the municipal rehabilitation services were not necessarily qualified to meet specific rehabilitation needs. *James* emphasized the importance of individual professional and specialist competence in rehabilitation by referring to his personal experience:

James: Well, I find that the competence of the therapists is a very important point. I notice a big difference in someone who really knows my illness, my situation…. For example, a general physical therapist is not necessarily sharp enough to provide good rehabilitation for me and my illness.

Third, for a rehabilitation team to function effectively, there is a need for teams with complementary qualifications and competence in multi‐professional collaboration. In addition, comprehensive information regarding the accessibility of multi‐professional teams in the municipality must be available to the service users:

Iris: I also think that multi‐professionality is important. That is, multi‐professional competence and treatment, but I need to know that this exists…. I experience … [the services] as quite fragmented; which possibilities are there, which services are provided?

It is notable that the user representatives incorporated personal experiences into their contributions to the discussion. When raising the issue of competence, they again brought in the tensions around what can be expected from specialist services in contrast to generalist municipal services.

### 3.4 Person‐Centeredness

Person‐centeredness was perceived as essential on several levels within the rehabilitation context. Participants' perspectives encompassed the individual patient–therapist interaction which included the family context, and person‐centeredness in institutional and political settings. Furthermore, rehabilitation organizations can support their individual members by assisting them in gaining access to necessary services.

At the micro level, rehabilitation was underscored as a shared decision‐making process that requires the involvement and commitment of both parties – the professionals and the patients. The role of personal attitudes and efforts in the rehabilitation process was emphasized:

Ava: I think that if you are going to get a good stay, good rehabilitation, it is primarily about your own attitude towards the rehabilitation, trying to do your best, and as I have experienced it…. one must participate and be active. Join exercise sessions, lectures, yes, and not least be involved in the social activities.

Patient‐centeredness was also important at an organizational meso level, particularly in the recruitment and requirement of user representatives with patient experience. Representatives with first‐hand patient experience would be better advocates than those without patient experience when contributing to the provision of appropriate health and rehabilitation services for the patient group they represent:

Jerome: It is we who know where the shoe pinches, not the ones who think they now. It is important that we bring in users with patient experience, that no bureaucrats come in and tell us what kind of needs we have.

It was highlighted that people without personal experience cannot adequately identify the needs of the users. However, participants also reflected on whether having patient experience by itself qualified a person to perform the functions of a user representative on an organizational level.

An additional view on service user involvement was presented by a representative from an organization that runs its own rehabilitation services. For this organization and the group it represents, user participation extends into clinical work.

Ida: We are actively involved in the service provision at the center. They are two, three even four rehabilitation assistants at each course which is really important, because the people coming to the course are led by example.

User involvement takes on a different meaning when the user organization owns the rehabilitation service, even if the service is predominantly financed by governmental funding. User organization ownership appears to carve a space for actively engaging experienced users in the provision of services.

### 3.5 System and Governance

Rehabilitation is understood as a trajectory across institutional settings and administrative levels that present opportunities for creating a stronger user voice.

James: I see it as very challenging, but also desirable if user participation could take place in some sort of forum across the organizational levels so that one could use the patient voice in the trajectory across levels… It would have to be a forum [for this] and [to succeed] it must be with some permanent representatives.

When user representation is confined to singular organizational levels, users' influence in achieving seamless services across levels is limited. A forum dedicated to this purpose with stable user participation could facilitate the inclusion of the patient voice across all levels of the rehabilitation trajectory.

In line with James' point above, the participants had concerns and differing experiences regarding their function as representatives in rehabilitation organizations. Some described being included and respected on an equal level with the other stakeholders and board members. Others described experiences of being present, but that their opinions and suggestions were not being fully taken into consideration or included in the meeting minutes. Iris reflected on what the organizations seek to achieve with the representatives on user councils, and how representatives should proceed to ensure they have an impact on the organization and provision of rehabilitation services:

Iris: At [rehabilitation institutions] we have representatives on the user councils… They do not discuss the services, and may not make any input or make suggestions … So, I think we are unclear about what we want with user participation in the institutions.

While user representatives serve as a link between patients and the management, the primary role should be as partners providing input on the content, organization and development of rehabilitation services. Hannah who had full rights as a member of a hospital board, did point to a positive change over the years in that the user representatives now are members of quality committees, patient safety committees and ethics committees.

Finally, a political turn in user participation was described by the representatives, characterized as an awakening and empowerment of user representatives and their organizations over time. This has led to a transition from primarily ensuring access and rights to rehabilitation for their patient groups at an individual level to voicing needs and opinions to the management of rehabilitation institutions at an organizational level and becoming political agents that influence policymakers and agenda‐setters:

Hannah: I see that these user organizations work much more politically. They have meetings in the Parliament, meet at different arenas where political decisions are being made, and that is where they must be, there where the money is allocated.

The user representatives described a balancing act of being close to the members' needs at the individual (micro) and organizational (meso) levels and serving as stakeholders giving voice to governance issues at the institutional and political (macro) levels.

### 3.6 Modes of User Representation and Contribution

The user representatives had disparate views and experiences regarding their function as user representatives at the organizational and political levels. Representing the interests of people with disabilities can require a challenging balance between exerting real influence and, like for *Neil*, avoiding becoming a hostage in decision‐making where the service users are in a minority position. In this respect, perspectives varied on whether adopting an external advisory position rather than an internal board member position would be more beneficial for the user group they represent:

Neil: The board is a collegium…. Then you can be stuck as a hostage. The advantage for me is that when I go in as an advisor, I have the right to speak and defend my position, but not the right to vote… Then you are free afterwards to say: “This is not the advice I have given”.

Another position was voiced by *Ellen*, who had not experienced being a hostage. *Ellen* emphasized that despite disagreements and lively discussions in board meetings, cooperating to reach a common platform and finding solutions by working from inside the boards and councils was necessary. The possibility of a middle position was also suggested, allowing user representatives to have their dissent noted in the minutes to avoid being disregarded in meetings.

A key question was how to fight tokenism. An obvious solution was to change the way boards work with user representatives. *Ralph* pointed to the need for organizations for providing training to qualify confident user representatives:

Ralph: Even if we have user representatives in almost all the [municipal] committees, this does not mean that we have an impact. I have a feeling that they were recruited because projects and committees must have a user representative; however, they have no influence. So, there is a great responsibility on the organizations to train good user influencers.

*Ralph* points out that the role of user representatives is in danger of being tokenistic. Interestingly, *he* did not suggest that the municipal committees should change their attitudes toward user representatives, but rather that representatives should become qualified to assume non‐tokenistic positions in committees. This included organizations taking responsibility through training and peer support for qualifying competent user representatives in conveying the organization's priorities when participating in local, regional or national committees.

## 4 Discussion

In this focus group study, the perspectives of representatives from user organizations for people with disabilities regarding rehabilitation service organization and provision were explored with the meso level and its connections with macro and micro levels as a theoretical background and analytical lens [3]. Six core themes were developed, which, though presented as separate themes with sub‐themes, represent interrelated topics of importance in rehabilitation service and provision.

### 4.1 Addressing Important Aspects of High‐Quality Rehabilitation Services

High‐quality rehabilitation services should be effective, safe and secure, coordinated, and characterized by continuity, user involvement, and efficient resource utilization [9, 29, 30]. Our findings on the key aspects of rehabilitation services align with challenges in rehabilitation service delivery identified by the WHO, which include integration of services into and between the administrative levels of health systems, access to multidisciplinary professionals, availability at community and hospital levels, and provision of specialized rehabilitation for people with complex needs [4]. Moreover, accommodating individual needs at a micro level and aligning these needs with the organization of service provision at the meso level is not straight forward. Health systems do not necessarily cater to the needs of individual user groups, which was expressed in the differing views on municipal versus specialized services and competence.

User representatives are situated in their diagnostic group and speak out for the diagnostic group they represent regarding access to services. Most notably are people suffering from musculoskeletal disorders encountering a more restricted access to specialized rehabilitation than other patient groups. This was perceived by the participants as a devaluation of their service user group in the organization and provision of rehabilitation services. Related to the WHO Rehabilitation 2030, Briggs et al. state that the burden of musculoskeletal disorders are not adequately recognized, and resource allocation is, thus not prioritized in accordance with the disease burden [31].

How the services are organized and provided was the predominant focus when services at the meso level were addressed. However, the user representatives' reflections shifted between the micro, meso and macro levels as interconnected components of their experience. Thus, the discussions also concerned issues such as governmental policy shifting the primary responsibility of rehabilitation provision for more common disorders to the municipalities [32, 33].

The municipal turn in Norwegian health services was problematized on several occasions during the interviews [32]. The user representatives consistently utilized personal experiences as a micro‐level starting point for the discussion of broader issues. A comparable finding was reported by Solvang et al. in a study of user involvement in a research project; the user representatives activated their personal biographies in their contributions to the running of the research project [28]. Both personal experiences and professional competences gained importance. Vaagan et al. pointed out that user involvement extends beyond clinical issues and encompasses engagement in health policy issues [34].

Effective rehabilitation often includes return to work as a goal, as absence from remunerative work is related to societal and individual costs [35, 36]. However, the paradigm of work as a primary rehabilitation goal was contested, as potentially contradicting the users' individual goals given their state of health and quality of life [37]. Moreover, the assumption that return to work should influence access to specialized rehabilitation was criticized, pointing to a potential incongruity between micro‐level rehabilitation guidelines emphasizing individual goals and shared‐decision making, which may not include return to work, and macro‐level health service and labor policies. Eriksson highlighted a possible challenge in the consensus rhetoric between policymakers and the user movement as it can mask fundamental disagreements, such as those revealed in this study over dilemmas in work‐life participation as a key goal for people with musculoskeletal disorders [24].

The importance of providing appropriate and timely services that meet the users' sense of urgency regarding their acute and long‐term needs was underscored in this study. What emerged was the need for a balance between more generic rehabilitation services at the municipal level and access to diagnosis‐specific follow‐up at the specialist level. This was followed by a suggestion to form an institutionally broad user forum that spans administrative levels from specialized to municipal services. Such a forum was proposed to significantly enhance the user voice and person‐centeredness at the organizational meso level and align with service coordination efforts. This could empower user representatives as stakeholders with an impact on the organization and provision of rehabilitation services at the meso level. Such a forum would emphasize the importance of rehabilitation as a process that requires a seamless transfer of competence and information across administrative levels [38]. Ensuring safe and secure healthcare services that prioritize patient‐centeredness [29] necessitates providing users with predictability. This requires comprehensive information‐sharing regarding diagnosis, prognosis, and the rehabilitation process across different institutional settings and administrative levels [39]. The user representatives emphasized the importance of predictability both from their personal experiences and during organizational‐level interactions where they had experienced that the system could fail to recognize the service needs of the users they represent. Vaagan et al. referred to this interconnection between the micro and meso levels as necessary to identify and understand the users' positions [34]. Andreassen described the position of involved users at the level of individual recovery as a sharing of their lived experience serving as co‐service providers, role models and conveyers of coping experiences at the meso level [40]. There remains a need for knowledge regarding the effectiveness and authenticity of user participation and shared decision‐making interconnecting the micro and meso levels [9, 41].

### 4.2 User Participation and Influence at the Organizational Meso Level

The user representatives had differing experiences regarding the level of impact in their positions. On the one hand, some described a feeling of being coerced into a role that lacked control or influence. This is in line with the study by Sagen et al. on patient engagement at the organizational level, in which a majority of the respondents were recruited from patient advisory boards [12]. Despite expressing satisfaction with the way their participation was organized by the institutions, they perceived their impact on institutional decisions as limited. On the other hand, some representatives who asserted their role and function as user representatives with confidence and gained acceptance for not being observers or advisors only.

User participation is embedded in the structures that support rehabilitation services from the individual to the policy level [41], and user organizations aim to have an impact and achieve benefits for their user groups. However, diverse perspectives on the impact and challenges of service user influence were revealed, and the extent of influence was questioned. The users' level of influence varied, and the impact of public participants' experiential knowledge in boards and councils can be questioned [41, 42].

As members of advisory bodies, user representatives are partners in co‐governance [40]. However, the possibility of co‐optation, where councils and boards incorporate users to manage threats and ensure alignment with organizational interests, risks disconnecting representatives from the members they represent [24]. The dilemma between maintaining integrity by taking an advisory position only versus a decision‐making position underscores the complexity of user influence. The former leaves representatives outside the decisional processes, while the latter enables responsibility for decisions and voting alongside other board members. Hence, there was a conflict between avoiding the position as a hostage and striving for a position as a respected actor representing the users' interests, and thus, being part of majority decisions.

A discrepancy appears to exist between the stated policy goal of delivering patient‐centered and equitable services emphasizing users as the source of control [29], and its actual implementation. The user representatives described organizational (meso) level strategies to fight tokenism and empower their members as user participation should go beyond tokenism and be on a partnership level, entailing real influence [42]. Strategies include intra‐organizational training of user representatives to enable their competent advocacy for members' needs and positions in service provision. Fighting tokenism necessitates user representatives' competence and assertiveness about their mandate, roles and participation in political processes. Yet, Beresford et al. highlight the risk of other stakeholders devaluing the users' experiential knowledge leading to tokenism at the meso and macro levels [43].

Similar to Morrison et al.'s study the user representatives in this study, experienced that their opinions and suggestions were not fully taken into consideration [41]. This experience is consistent with Sagen et al.'s findings regarding patient advisory boards in rehabilitation institutions, where a substantial proportion (36%) of the user representatives were unaware of their voting rights [12]. This suggests that institutions fail to adequately inform user representatives about their voting rights. Sagen et al. refer to this as a challenge in overcoming the glass ceiling for genuine user influence [12].

### 4.3 Strengths and Limitations

A strength of this focus group study is the inclusion of a diversity of user organization representatives of both genders, and from both local and central divisions of the organizations. Furthermore, we conducted a final meeting as part of the validation of the study, during which the representatives recognized the themes developed in the analysis and provided additional details to strengthen the findings. However, given the multiplicity of user organizations for people with disabilities, a bias in the recruitment of user organization representatives might have influenced the results. There might have been a self‐selection to the study where user organizations that perceive their user group as relevant to the study or have more resources for research activities may have chosen to participate. Furthermore, the user representatives shared their experiential knowledge when participating in this study; however, whether user representatives' personal experiences are perceived as legitimate in other participatory arenas has been questioned [12, 41]. An additional potential limitation of the study was the online setting of the focus groups. However, traveling long distances to in‐person meetings may have been a burden for participants with disabilities, which could have limited participation.

## 5 Conclusion

The service user representatives demonstrated tension and complexity in their meso‐level engagement in the provision and organization of rehabilitation services. The challenges pertained to both the general quality of services and those specific to the user groups they represent. They experienced challenges in participating in and exerting influence on boards and councils. Empowering user representatives through training was important to fight tokenism. To fill the role as user representatives at the meso level on the organization of rehabilitation services, both the micro level of personal and peer experiences and health policy regulations and reform at the macro level are at play.

## Author Contributions

**Helene Lundgaard Søberg:** conceptualization, investigation, writing–original draft, methodology, validation, formal analysis, project administration, writing–review and editing. **Per Koren Solvang:** writing–original draft, validation, formal analysis, writing–review and editing. **Nada Andelic:** conceptualization, writing–original draft, methodology, validation, writing–review and editing. **Cecilie Røe:** conceptualization, writing–original draft, methodology, validation, project administration, writing–review and editing. **Marit Kirkevold:** conceptualization, investigation, writing–original draft, methodology, validation, project administration, formal analysis, writing–review and editing.

## Conflicts of Interest

The authors declare no conflicts of interest.

## Data Availability

The data that support the findings from the focus group interviews is not available due to data protection regulations.
